# Cross-cultural adaptation and validation of the Arabic version of the foot function index in patients with chronic lateral ankle instability

**DOI:** 10.1186/s13047-022-00527-6

**Published:** 2022-03-12

**Authors:** Mohammed I. Amri, Msaad M. Alzhrani, Ahmad D. Alanazi, Mazen M. Alqahtani, Faizan Zaffar Kashoo

**Affiliations:** 1 Department of Medical Rehabilitation, Physical therapy department, King Salman Hospital, Ministry of Health, Riyadh, 12769 Kingdom of Saudi Arabia; 2 Department of Physical Therapy and Health Rehabilitation, College of Applied Medical Sciences, Majmaah University, Majmaah, 11952 Saudi Arabia

**Keywords:** Cross cultural adaptation, Foot function index, Lateral ankle sprain, Foot function index Arabic version, Chronic lateral ankle instability

## Abstract

**Background:**

The English version of the Foot Function Index (FFI) is a reliable and valid tool for measuring pain and functional instability due to chronic lateral ankle instability (CLAI). However, its use among Arabic speakers with CLAI is limited because of the unavailability of the Arabic version of the FFI (FFI-Arb). This study aimed to translate, cross-culturally adapt and validate the FFI from the original English version into Arabic.

**Methods:**

The FFI questionnaire was translated using the Beaton guidelines. Two-hundred-and-thirty native Arabic-speaking participants with CLAI were recruited from different physiotherapy clinics in Saudi Arabia. The convergent validity of the FFI-Arb was tested using the Spearman correlation with the Arabic version Cumberland ankle instability tool (CAIT-Arab). Test-retest reliability was tested among 92 participants who completed the form again after seven days.

**Results:**

Two-hundred-and-thirty participants were enrolled (mean age = 32.09, Standard deviation (SD) = 8.64 years old). There was excellent internal consistency for the three subscales of FFI: pain (0.95), disability (0.97), and activity limitation (0.86), as for the total score (0.98). Convergent validity was analyzed by Spearman rank correlation between the new translated versions of FFI-Arb and CAIT-Arab. The total FFI-Arb and CAIT-Arab scores were moderately correlated (*rho* = − 0.569; *p* < 0.001). Subscales of FFI-Arb, such as pain, disability, and activity limitation, were also moderately correlated with CAIT-Arab (*rho* = − 0.565, *rho* = − 0.561, *rho* = − 0.512; *p* < 0.001). The construct validity was confirmed by principal component analysis (factor analysis) showing a three-factor structure (eigenvalue 1) of FFI-Arb with a total variance of 77.3%. Test-retest reliability was excellent for the total score of the FFI-Arb and all its subscales (interclass correlation coefficient = 0.984–0.999).

**Conclusions:**

The FFI-Arb is a reliable and valid tool for Arabic-speaking patients with CLAI. The FFI-Arb can be utilized in hospitals and clinics in Arabic speaking countries.

**Supplementary Information:**

The online version contains supplementary material available at 10.1186/s13047-022-00527-6.

## Background

Lateral ankle sprain (LAS) is a common musculoskeletal injury among individuals who participate in recreational physical activities and competitive sports [1–4]. LAS is associated with a significant increase in medical costs [5] and restricted mobility [6]. Ankle sprains account for significant healthcare expenditure in the United States and can lead to chronic pain, physical disability, and functional limitations [7]. A study reported that approximately 70% of the general population suffers from ankle injuries in their lifetime [8]. There is a high incidence of LAS during recreational and competitive sports, with an incidence of 2.15 per 1000 in the United States and 6.09 per 1000 in the United Kingdom [9, 10]. The prevalence of LAS in the general population is reported to be high [8]. A recent study conducted in Saudi Arabia among male high school students reported a 14 to 34.7% prevalence of ankle sprain [11].

In 2001, Hertel et al. [12] proposed a model consisting of mechanical insufficiency and functional insufficiency contributing to chronic ankle instability (CAI). Later in 2011, Hiller et al. [13] proposed an extension of the Hertel model of CAI. Individuals with CAI were categorised into 7 clinical subgroups based on mechanical instability, perceived instability and recurrent sprains. Sports activities such as volleyball, football, and basketball, which require players to perform running, jumping, and cutting activities, are reported to cause frequent ankle injuries [8]. Approximately 60% of CAI in players arise from contact or direct trauma [14]. Usually, ankle sprains occur during the transformation from the non-weight-bearing position to the loaded position [15]. Most ankle sprains cause injury to the lateral ligament complex [16]. CAI develops between six weeks and 18 months post ankle sprain in 55 to 70% of individuals with incomplete recovery [17]. There is inconsistency in the research literature about terminology to describe the chronic ankle sprain as functional instability, residual instability, and chronic instability. For this article the term chronic lateral ankle instability (CLAI) will be used in the manuscript to denote the repetitive nature of ankle sprain.

The foot function index (FFI) questionnaire [18] and four other tools for the foot and ankle [19–22] are the only tools that have been widely used in clinical settings. The FFI is a self-reported instrument used to assess foot and ankle function in terms of pain, activity limitation, and disability following ankle or foot injury [18]. The FFI consists of three dimensions: pain (9 items), disability (9 items), and limitation of activity (5 items). It is reported to be a feasible tool, easy to calculate, and takes less than 5 min to answer [23, 24]. Additionally, it is reliable and valid in other foot and ankle conditions such as CLAI and Achilles tendinopathy [25, 26]. However, its use among Arabic speakers with CLAI is limited because of the unavailability of the Arabic version of the FFI. Therefore, the aims of this study was to cross-culturally adapt and translate the FFI questionnaire from the original English into Arabic language. The second aim was to test the validity and reliability of the Arabic translated version of the FFI among patients with CLAI in Saudi Arabia.

## Methods

### Design

This was a cross-sectional study. Formal permission was obtained from the original developer to translate and cross-culturally adapt the Arabic version of the FFI questionnaire [18]. The protocol was approved by the central institutional review board of the Ministry of Health in the Kingdom of Saudi Arabia (NO: 20-184 M). Participants were asked to sign an informed consent form. The participants were free to discontinue the study at any time without reason.

### Setting

**Fig. 1:**
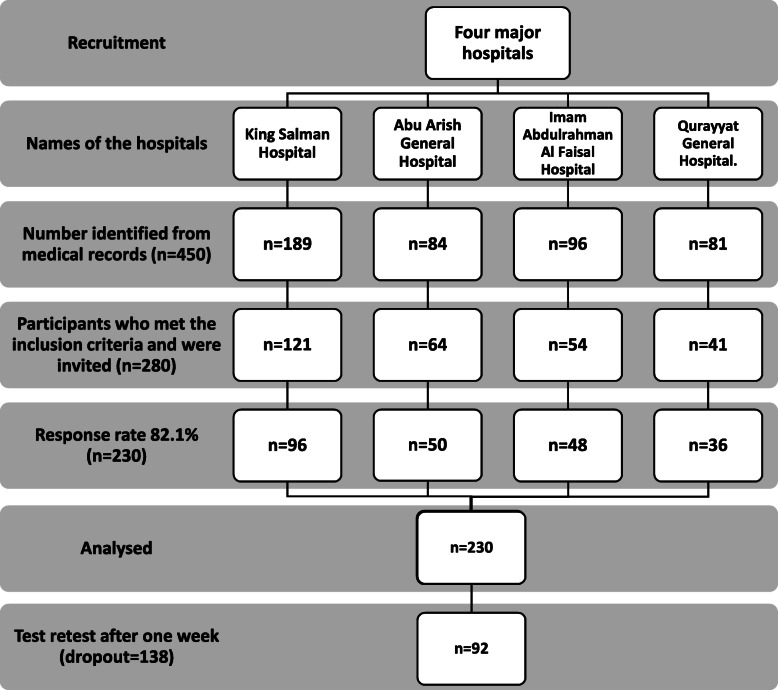
Flow Chart of the sample population, respondents, inclusion, and analysis

Two-hundred-and-thirty native Arabic-speaking participants with CLAI were recruited from different physiotherapy clinics located in the Kingdom of Saudi Arabia. The retrospective medical records for the last 5 years were electronically searched in four major hospitals in Saudi Arabia: King Salman Hospital, Imam Abdulrahman Al Faisal Hospital, Abu Arish General Hospital, and Qurayyat General Hospital (Fig. 1).

### Sample size calculation

This study recruited 230 subjects diagnosed with CLAI based on the guidelines for the respondent-to-item ratio ranging from 10:1 (230 respondents for a 23-item questionnaire) [27].

### Inclusion and exclusion criteria

Participants were aged over 18 years, consented to participate in the study, and were recruited between November 2020 and January 2021, from the Arab population in Saudi Arabia with history of CLAI more than 12 months and persistence of ankle “perceived instability.” Participants were native Arabic speakers with the ability to read and understand Arabic. Additionally, standard inclusion criteria endorsed by the International Ankle Consortium for patients falling into the category of chronic ankle instability were used [28]. Subjects with neurological disorders, mental illness, cognitive impairments, other lower extremity disease, and associated foot deformities were excluded.

### Questionnaires

The questionnaires contained brief descriptions of the study with inclusion and exclusion criteria, consent form, and demographic data such as sex, age, weight, and height. In addition, it included the injured leg, dominant leg, level of sports activities, and duration of injury. Subsequently, 230 participants filled out the newly translated Arabic version of the FFI questionnaire (FFI-Arb) and the previous translated Arabic version of the Cumberland Ankle instability tool (CAIT-Arab).

The original English version of the FFI questionnaire is a self-reported scale used to measure ankle function after ankle injury [18]. The questions in the FFI questionnaire were categorized into three subscales: pain, disability, and activity limitation [18]. The pain subscale consists of nine questions related to foot pain in different situations. The disability subscale consists of nine questions related to difficulty performing various functional activities because of ankle injuries. Activity limitation consists of five questions related to limitations in various activities due to ankle injury. The participant scored each question based on a 0–10 visual analog scale, with a higher score indicating the worst pain or disability. Scores from each subscale were summed and presented as percentages. The overall scores of the three scores were divided by 170 to obtain a total average score.

The CAIT-Arab is a valid, reliable, and responsive scale for measuring CLAI in Arabic-speaking individuals [29]. The CAIT consists of nine items with several answers that evaluate various components of CLAI, including ankle pain, perceptual instability during daily and physical activity, and the ankle’s sensitivity to events of giving way. The nine items add up to a total score that ranges from 0 to 30, with lower values indicating more severe instability and 30 being the highest attainable stability score. To identify patients with CLAI, the initial study used a cut-off score of 27. The rationale to use CAIT to evaluate convergent validity of FFI was that the tool is freely available in Arabic validated version [29], measure a similar construct and is widely used in clinical settings. The Arabic version of the CAIT has been reported to have excellent internal consistency (α = 0.92), reliability (interclass correlation coefficient [ICC] 0.75–0.98), convergent validity with lower extremity functional scale (ρ = 0.67), and 1-factor solution explaining 63.8% of the variance [29]. The questionnaire consists of nine items measuring functional ankle instability, with scores ranging from 0 to 30, with lower marks suggesting more extreme instability [29].

To establish the test-retest reliability of the FFI-Arb, participants were invited again to fill the online FFI-Arb.

### Translation and cross-cultural adaptation

The translation and cultural adaptation of the FFI followed the method described by Beaton et al. [30]. The six steps of this guideline include forward translation, synthesis, backward translation, expert community analysis, pretesting, and expert community evaluation of this process.

First step, Forward translation: This forward translation was undertaken by two bilingual native Arabic translators who translated the original FFI English version into an Arabic version. The original FFI version was translated into Arabic by an informed translator with a background in physiotherapy (T1), who is a native Arabic speaker and fluent in English. The original FFI version was translated into Arabic by a second blinded translator with no medical experience (T2), who is also a native Arabic speaker and proficient in English.

Second step, Synthesis of the translations: (Last translated – T12), to extract the final translated version using the findings of the translated versions of both T1 and T2. Both T1 and T2 translators and a moderator discussed the details of both versions and addressed any inconsistencies between the two translated versions and eventually created a final version of Arabic FFI (T12).

Third step, Back Translation: The T12 version was translated back to its original version (English) by two back-translators independently who were fluent in both English and Arabic languages (bilingual), with English as the dominant spoken language (English) (BT1, BT2). To minimize the risk of bias during the translation process, both BT1 and BT2 translators were blinded to the original version of the FFI. After completing each translation process, each translator was given a brief report.

Fourth step, Expert Committee Review: Committee members comprised the translators (forward and back translators), language professionals, principal investigators, and methodologists who all met to discuss all translated versions to approve and create a pre-final Arabic FFI version.

Fifth step, Test of the Pre-Final Arabic FFI Version: A pre-final (pilot test) was performed with 23 participants with a history of lateral ankle sprain to check whether the FFI questionnaire was understandable for them all and to collect their feedback and comments. After participants completed the pre-final questionnaire, their feedback was included in Appendix-1.

Sixth step, Expert Committee Approval: Expert members met to resolve all participants’ comments and established the final version of the FFI Arabic version to examine the validity of the scale (Appendix-2).

### Statistical analysis

Statistical analysis of the data was performed using the Statistical Package for Social Sciences for Windows, version 26 (SPSS Inc., Chicago, IL, USA). Statistical significance was set at *P* < 0.05. Internal consistency for the FFI-Arb was examined using Cronbach’s alpha (CA). The CA values ranged from 0 (no internal consistency) to 1 (perfect internal consistency). The grading system described by George et al. [31] was used for internal consistency as Excellent (equal to or more than 0.90), Good (equal to or more than 0.80), Acceptable (equal to or more than 0.70), Questionable (equal to or more than 0.60), Poor (equal to or more than 0.50), Unacceptable (less than 0.50).

The test-retest reliability for FFI was tested using the interclass correlation coefficient (ICC) [32]. ICC values below 0.5 were considered weak reliability, values between 0.50 and 0.70 were considered moderate reliability, values between 0.75 and 0.90 were considered good reliability, and values above 0.90 indicated excellent reliability.

Construct validity was evaluated using principal component analysis with varimax rotation. Convergent validity was examined using Spearman’s coefficient correlation between scores of FFI-Arb and CAIT-Arab, which were interpreted as flows: values from 0.00 to 0.19 was very week correlation, from 0.20 to 0.39 was weak correlation, between (0.40 to 0.69) was moderate correlation, from (0.70 to 0.89) was a strong correlation, and from (0.90 to 1) was very strong correlation.

Ceiling and floor effects were evaluated by analyzing the extremes of scoring among participants. If more than 15% of participants scored extreme scores, then the scale was said to have either a ceiling effect (high scores) or floor effect (low scores).

## Results

### Participants

Two-hundred-and-thirty participants completed both the FFI-Arb questionnaire and CAIT-Arab at baseline. Ninety-two completed the FFI-Arb questionnaire again after seven days to examine the reliability.

Total number of participants (*N* = 230) mean age was 32.09, Standard Deviation (SD) = 8.64 years (Table 1). The majority of the participants were men (*N* = 179). The mean of body mass index for 230 participants was 28.87 kg/m^2^, SD = 22.60 (Table 1). Sixty percent of participants (*N* = 137) suffered from right ankle injury with right-leg dominant, whereas 40% of participants (*N* = 93) suffered from left leg injury with left-leg dominant.

**Table 1:** Demographic Characteristics of Participants (Age, Weight, Height, injury date) *N* = 230, *N* = 92

Number of participants	***N*** = 230	***N*** = 92
	Mean ± Std. Deviation	
**Gender (male/female)**	179/51	67/25
**Age (years)**	32.09 ± 8.642	32.99 ± 8.599
**Body Mass Index (kg/m**^**2**^**)**	28.87 ± 22.60	32.42 ± 32.79
**When did the injury happen? (weeks)**	160.91 ± 187.80	158 ± 170

The majority of the participants (33%) reported playing sports activities daily, followed by 26% of the participants who reported playing sports often, followed by 29% of the participants reporting playing sports sometimes, 9% of participants reported playing sports rarely, and only 3% of the participants reported that they never played any kind of sports.

The characteristics of the 92 participants who answered the FFI-Arb twice are shown in Table 1; the mean age = 32.99 years, SD = 8.599. Seventy-three percent of the subjects (*N* = 67) were male while 27 of them were female (*N* = 25). The number of participants with a history of right leg injury was 58% (*N* = 53), while that of the left leg was 42% (*N* = 39). The percentage of participants with dominant right leg (82%, *N* = 75) was higher than that of participants with left leg dominance (18%, *N* = 17). Regarding the sport activity levels of the participants, 24% of the participants played every day, 34% of the participants often played sports, 30% of the participants sometimes played sports, 9% rarely played sports, and 3% never played any kind of sports.

### Convergent validity between the FFI-Arb and CAIT-Arab questionnaire

With the scoring system, the average total FFI-Arb score of the participants was 51.70, SD = 48.06. Averaged subscales of pain, disability, and activity limitation were 20.91, SD = 18.78, 23.40, SD = 22.62, and 7.39, SD = 9.07, respectively. The average and SD for FFI-Arb scores are summarized in Table 2. The average total CAIT was 18.48, SD = 5.53. The averages and SDs for CAIT-Arab scores are summarized in Table 3.

**Table 2:** Description of the items of FFI-Arb

FFI-Arb Questionnaire	Mean ± Std. Deviation
FFI-1	3.52 ± 2.84
FFI-2	2.37 ± 2.84
FFI-3	2.96 ± 2.89
FFI-4	2.59 ± 2.89
FFI-5	2.62 ± 2.60
FFI-6	2.27 ± 2.58
FF-7	0.71 ± 1.74
FF-8	0.61 ± 1.57
FF-9	3.27 ± 2.79
**Pain Total**	**20.91 ± 18.78**
FF-10	1.89 ± 2.46
FF-11	2.37 ± 2.69
FF-12	2.87 ± 2.92
FF-13	2.89 ± 2.93
FF-14	2.66 ± 2.81
FF-15	3.42 ± 3.15
FF-16	1.85 ± 2.48
FF-17	2.09 ± 2.54
FF-18	3.37 ± 3.15
**Disability Total**	**23.40 ± 22.62**
FFI-19	1.98 ± 2.50
FFI-20	1.63 ± 2.46
FFI-21	2.80 ± 2.67
FFI-22	0.52 ± 1.82
FFI-23	0.46 ± 1.72
**Activity Limitation Total**	**7.39 ± 9.07**
**FFI-Arb Total**	**51.70 ± 48.06**

**Table 3:** Description of the items of the Cumberland Ankle instability tool Arabic version (CAIT-Arab)

CAIT-Arab	Mean ± Std. Deviation
CAIT**-**1	3.47 ± 1.44
CAIT**-**2	2.28 ± 1.42
CAIT**-**3	1.83 ± 1.07
CAIT**-**4	2.27 ± 0.87
CAIT**-**5	1.38 ± 0.70
CAIT**-**6	2.08 ± 1.09
CAIT**-**7	2.82 ± 1.16
CAIT**-**8	1.54 ± 1.09
CAIT**-**9	0.80 ± 1.09
**CAIT Total**	**18.48** ± **5.53**

To examine the convergent validity of the FFI-Arab, Spearman’s rank-order correlation coefficient test was used because the data were not normally distributed, as determined by the Shapiro-Wilk test (total FFI-Arb = 0.897, *p* < 0.001). The total FFI-Arb and CAIT-Arab scores were negatively and moderately correlated (*rho* = − 0.569; *p* < 0.001). In addition, the correlation of the subscales of FFI-Arb, such as pain, disability, and activity limitation, was negatively and moderately correlated with CAIT-Arab (*rho* = − 0.565; *p* < 0.001), (*rho* = − 0.561; *p* < 0.001), and (*rho* = − 0.512; *p* < 0.001), respectively (Table 4).

**Table 4:** Convergent validity (*N* = 230)

FFI Questionnaire			CAIT-Arab Total
**Spearman’s rho**	**Pain**	Correlation Coefficient	−.565^**^
		Sig. (2-tailed)	< 0.001
		N	230
	**Disability**	Correlation Coefficient	−.561^**^
		Sig. (2-tailed)	< 0.001
		N	230
	**Activity limitation**	Correlation Coefficient	−.512^**^
		Sig. (2-tailed)	< 0.001
		N	230
	**FFI-Arb Total**	Correlation Coefficient	−.569^**^
		Sig. (2-tailed)	< 0.001
		N	230

### Construct validity of the FFI-Arb

The construct validity of the FFI questionnaire was estimated using principal component analysis (factor analysis) with varimax rotation (Table 5). The Kaiser–Meyer Olkin measure of sample adequacy was tested using Bartlett’s test of sphericity and was found to be statistically significant (6300.154; *p* = 0.001). The total variance was 77.3%. The analysis found three factor structure of questionnaire; Factor 1 included first 19 items of the questionnaire (64.85% of variance), Factor 2 included item 20 and 21 (7.89% of variance), Factor 3 included item 22 and 23 (4.56%) (Table 5).

**Table 5:** Factor Loading with 3 factors (Rotated Component Matrix)

Item Number	Questions	Factor structure		
		Pain	Disability	Activity limitation
Item 1	Worst Leg Pain	.802	.161	.130
Item 2	Morning Leg Pain	.786	.230	.142
Item 3	Walking Leg Pain	.849	.165	.095
Item 4	Standing Leg Pain	.798	.254	.055
Item 5	Walking Shoes Pain	.845	.285	.040
Item 6	Standing Shoes Pain	.789	.313	.078
Item 7	Walking Orthotics Pain	.258	.861	.229
Item 8	Standing Orthotics Pain	.235	.874	.257
Item 9	End Day Leg Pain	.809	.288	−.076
Item 10	Difficulty Walking In House	.829	.277	.164
Item 11	Difficult Walking Outside	.848	.271	.100
Item 12	Difficult Walking 800Meters	.885	.150	.184
Item 13	Difficulty Climbing Stairs	.855	.135	.259
Item 14	Difficulty Descending Stairs	.832	.175	.309
Item 15	Difficulty Standing Tip Toe	.829	−.030	.202
Item 16	Difficulty Getting Up Chair	.791	.117	.237
Item 17	Difficulty Climbing Curbs	.850	.145	.249
Item 18	Difficulty Walking Fast	.857	.126	.266
Item 19	Stay Inside All Day	.767	.149	.294
Item 20	Stay In Bed	.710	.336	.336
Item 21	Limited Activities	.812	.190	.201
Item 22	Use Assistive Device Indoor	.202	.167	.881
Item 23	Use Assistive Device Outdoor	.164	.259	.843

Extraction Method: Principal Component Analysis

Rotation Method: Varimax with Kaiser Normalization

a. Rotation converged in 5 iterations

### Internal consistency of the FFI-Arb

CA was used to examine the internal consistency of the FFI-Arb. The FFI-Arb showed excellent internal consistency for the total scores of the FFI (0.98) and two subscales of pain (0.95) and disability (0.97). However, the internal consistency of the activity limitation subscale was good (0.86).

### Test-retest reliability

Out of 230, only 92 participants responded even after three consecutive (every week) reminders. The test-retest reliability was assessed 7 days after the initial assessment and was found to be excellent**.** The test-retest reliability was excellent for the overall FFI-Arb, and the average Interclass correlation coefficient (ICC) was 0.998, with a 95% Confidence interval (CI) (0.997, 0.999) (*p* < .001). In addition, the reliability of all three subscales was excellent. For the three subscales—pain, disability, and activity limitation—the average measure ICC was 0.996, with a 95% CI [0.994, 0.997] (*p* < .001), the average measure ICC was 0.999, with a 95% CI [0.998, 0.999] (*p* < .001), and the average measure ICC was 0.984 with a 95% CI [0.997, 0.999] (*p* < 0.001), respectively (Table 6).

**Table 6:** Intraclass Correlation Coefficient (ICC) of FFI *N* = 92

Item	Average measure	Confidence interval Lower Bound – Upper Bound	***P*** Value
**Pain**	0.996	0.994–0.997	< 0.001
**Disability**	0.999	0.998–0.999	< 0.001
**Activity limitation**	0.984	0.976–0.989	< 0.001
**FFI-Arb Total**	0.998	0.997–0.999	< 0.001

### Floor and ceiling effects

There was no ceiling or flooring effect for the FFI-Arb total score or any of its three subscales because no participants reached the highest or lowest score (Table 7).

**Table 7:** Floor and ceiling effect

FFI-Arb questionnaire	Floor	Ceiling
**Pain**	< 5.7	> 0
**Disability**	< 10.9	> 0
**Activity Limitation**	< 14.8	> 0
**Total FFI-Arb**	< 4.3	> 0

## Discussion

This is the first study to translate, adapt, and validate the English version of the FFI into Arabic for native Arabic speaking participants with CLAI. This study reports that the FFI-Arb has excellent reliability and internal consistency, moderate convergent validity, and no floor or ceiling effects.

The test-retest reliability of the FFI-Arb was assessed after a one-week interval. The test-retest reliability was excellent for the pain, disability, and activity limitation domains of the FFI-Arb. A research study conducted among 53 German-speaking patients with foot complaints reported excellent internal consistency of the German version of FFI [24]. The study also reported a high correlation between the German-version of FFI with a Short form health survey of 36 items [24]. Similar results have been reported in articles involving translation and cross-culturally adapted FFI questionnaires in Spanish [33], Thai [34], Brazilian Portuguese [35], Korean [36], and Chinese [37] reported good to excellent test-retest reliability. A study conducted among 35 Danish-speaking populations reported that the Danish version of FFI is a reliable and valid instrument for foot-related disorders [38]. However, the study reported that the Danish version of the FFI exhibited floor and ceiling effects. This may have occurred because of the small sample size [38].

This study found that the FFI-Arb is a reliable instrument among native Arabic-speaking patients with CLAI. The FFI-Arb demonstrated excellent internal consistency for pain and disability but had good internal consistency for activity limitation. These results are similar to previous findings that reported that the activity limitation domain had good internal consistency, while the pain and disability domains demonstrated excellent internal consistency [23, 24, 34, 39, 40]. However, few studies, such as the cross-cultural adaptation the Danish [38] and Chinese versions [32], have reported excellent internal consistency in all three domains of FFI.

The use of instruments not specifically designed to assess ankle injuries may influence outcomes. Therefore, a precise ankle assessment measurement was used, and this was the first study to use CAIT in the FFI validation process. The total FFI-Arb and CAIT-Arab scores were negatively and moderately correlated (*rho* = − 0.569). In addition, the correlation of the subscales of FFI-Arb, such as pain, disability, and activity limitation, was moderately correlated with CAIT-Arab (*rho* = − 0.565, *rho* = − 0.561, *rho* = − 0.512).

Our study reports moderate convergent validity of the FFI with CAIT. One of the possible reasons for moderate correlation was the difference in the number of items between FFI (23 items) and CAIT (9 items). Since pain, disability and activity limitation is a multidimensional construct, the items used to evaluate pain, disability and activity limitation are more comprehensive in FFI than in CAIT. Similarly, an FFI Spanish version (FFI-Sp) reported moderate to high correlations between the FFI-Sp with Foot Health Status Questionnaire and the visual analog scale, the EuroQol 5-D, visual, and the Short Form Health Survey (SF-12) [33]. A study conducted among 113 Persian-speaking individuals in Iran reported moderate convergent validity between the Manchester Oxford Foot Questionnaire and the Persian version of the FFI [40]. Similarly, a study conducted in Thailand among 97 Thai speakers with chronic ankle sprain reported moderate correlation of the Thai-version of FFI with the visual analog scale for Pain and Short Health Form-36 [23]. Another study conducted in Brazil among 50 participants with chronic ankle sprain, plantar fasciitis, and metatarsalgia reported a significant correlation between the Brazilian-Portuguese version of the FFI questionnaire with sub-scales of Short Health Form-36 (pain and social aspect) and all subscales of the Foot and Ankle Outcome Score questionnaires, except for “other symptoms” [35].

The current study did not demonstrate the floor or ceiling effects. Similarly, studies reported that there were no floor or ceiling effects for the FFI total score or any of its three subscales because none of the participants had reached the highest or lowest score [35, 38, 41].

There was high dropout rate in our study. The main reason of high dropout rates was unknown, however the most probable reason of high drop-out rate was Corona pandemic and lock-down during the time retest was required from participants in Saudi Arabia.

Future studies can be conducted to evaluate the other psychometric parameters of FFI, such as confirmatory factor analysis. The convergent validity can be further evaluated with scales measuring a similar construct. Since the validation of FFI included population with mean age of 32.09, it would be intriguing to further investigate the sensitivity of FFI in older population with CLAI.

### Limitation

Self-reported questionnaires have many drawbacks, such as response rate, understanding and lack of honest answers to sensitive questions; individuals may not be able to assess themselves accurately. However, the questions in the FFI-Arb were neutral and showed considerably reduced response bias. The response bias is further reduced by nature of the questions being non-guiding. The questionnaire contained 23 questions with relatively short questions, reducing the acquiescence bias. The random presentation of questions in the questionnaire reduced the question order bias. Only patients with CLAI were included in this study, which may limit generalizability to other ankle injuries. For this purpose, more diverse injuries should be included in future studies. Furthermore, this study did not report sensitivity to alternations, responsiveness, and error scores, which would be helpful for clinical decision making. Moreover, assessing the validity of the FFI against other Arabic ankle questionnaires would be beneficial. However, this research as yet still remains to be conducted. The dropout rate (*N* = 138) during the test retest was high. However, the number of responses (*N* = 92) was sufficient to analyze the test-retest reliability.

## Conclusion

FFI-Arb was simple and easy to understand among Arabic-speaking participants with CAS. The translated version contains Arabic dialects and terms that are commonly used in Arabic, and the questionnaire is culturally acceptable. The FFI-Arb questionnaire demonstrated excellent test reliability and construct validity for Arabic-speaking participants with CLAI. It can also be used in clinical practice and research.

## Supplementary Information

**Additional file 1:** **Appendix 1.**

**Additional file 2:** **Appendix 2.**

**Additional file 3:** **Appendix 3.**

## Data Availability

The data will be provided upon request to corresponding author.

## Abbreviations

FFI: Foot Function Index
CAS: Chronic Ankle Sprain
CAI: Chronic Ankle Instability
CLAI: Chronic Lateral Ankle Instability
CAIT: Cumberland Ankle Instability Tool
SD: Standard Deviation
N: Number
CI: Confidence Interval
ICC: Interclass Correlation Coefficient
CA: Cronbach’s alpha
