# Supplementary material for: Cross-cultural adaptation and validation of the Arabic version of the foot function index in patients with chronic lateral ankle instability
Source: J Foot Ankle Res. 2022 Mar 12;15:21. doi: 10.1186/s13047-022-00527-6 (PMC8917771; doi:10.1186/s13047-022-00527-6)
Supplement: Supplementary file 1 — Additional file 1: Appendix 1. [file 13047_2022_527_MOESM1_ESM.docx]

1. Question number 7 and 8. Pain walking with orthotics? And Pain standing with orthotics? Some participants asked about the meaning of orthotics and requested clarity for example did this refer to a brace, compression bandage, splint etc.
2. Question number 22 and 23 which stated that the “Use assistive device indoors” and use assistive device outdoors”. Some participants asked about the meaning of the term assistive device. Notes were added to the beginning of questions that assistive devices meant either walking stick or crutches.
3. Question number 7, 8, 22 and 23, If a participant stated that an action such as assistive device was not used or wearing an orthotic was not done, the participant had to leave the question blank. Some participants did not understand those questions, to clarify, Notes were added to the beginning of questions if they did not use assistive device or orthotics, keeping the question blank.
4. Question number 12 which is (difficulty walking 4 blocks?) This was subsequently modified into (difficulty walking 800 meters) after contacting the main author of the original FFI (Budiman-Mak) who defined one block is equal to 200 meters^13^.
