# Supplementary material for: Cross-cultural adaptation and validation of the Arabic version of the foot function index in patients with chronic lateral ankle instability
Source: J Foot Ankle Res. 2022 Mar 12;15:21. doi: 10.1186/s13047-022-00527-6 (PMC8917771; doi:10.1186/s13047-022-00527-6)
Supplement: Supplementary file 2 — Additional file 2: Appendix 2. [file 13047_2022_527_MOESM2_ESM.pdf]

Foot Function Index

Patient Name: ________________________________ Date:____________

This questionnaire has been designed to give your therapist information as to how your foot pain has affected your ability to manage in everyday life. Please answer every question. For each of the following questions, we would like you to score each question on a scale from 0 (no pain or difficulty) to 10 (worst pain imaginable or so difficult it required help) that best describes your foot over the past WEEK. Please read each question and place a number from 0-10 in the corresponding box.

No Pain __1 2 3 4 5 6 7 8 9 10__ Worst Pain Imaginable

Pain Subscale: How severe is your foot pain:

Foot pain at its worst?	
Foot pain in morning?	
Pain walking barefoot?	
Pain standing barefoot?	
Pain walking with shoes?	

Pain standing with shoes?	
Pain walking with orthotics?	
Pain standing with orthotics?	
Foot pain at end of day?	

Disability Subscale: How much difficulty did you have:

Difficulty walking in house?	
Difficulty walking outside?	
Difficulty walking 4 blocks?	
Difficulty climbing stairs?	
Difficulty descending stairs?	

Difficulty standing tip toe?	
Difficulty getting up from chair?	
Difficulty climbing curbs?	
Difficulty walking fast?	

Activity Limitation Subscale: How much of the time do you:

Stay inside all day because of feet?	
Stay in bed because of feet?	
Limit activities because of feet?	

Use assistive device indoors?	
Use assistive device outdoors?	

Score: ____/23**0 points**

النسخة العربية لاستبيان مؤشر وظيفة القدم (FFI-Arb)

تم تصميم هذا الاستبيان لكي يعطي اخصائي العلاج الطبيعي المعالج لك معلومات عن ألم قدمك وتأثيره على قدرتك للقيام بالنشاطات اليومية المختلفة. الرجاء الإجابة على كل الأسئلة.

ولكل سؤال نرجو منك تقييم إجابتك من ٠ (لا يوجد ألم أو صعوبة) إلى ١٠ (أقصى ألم ممكن تصوره او صعب جداً وغير قادر) وذلك لوصف مقدار ألم قدمك خلال الأسبوع الماضي.

الرجاء قراءة كل سؤال ووضع الرقم المناسب في المكان المخصص لذلك.

لا يوجد ألم	٠	١	٢	٣	٤	٥	٦	٧	٨	٩	١٠	أقصى ألم ممكن تصوره

**مقياس الألم:<br>ما مدى شدة ألم قدمك:**	**الدرجة**
١- ألم القدم في أسوأ حالاته؟	
٢-ألم القدم في الصباح؟	
٣-الألم عند المشي حافيًا؟	
٤-الألم عند الوقوف حافيًا؟	
٥-الألم عند المشي بحذاء؟	
٦-الألم عند الوقوف مرتديا الحذاء؟	
٧- الألم عند المشي مرتديًا جبيرة تقويم العظام؟ مثل الجبيرة والأربطة الضاغطة وغيرها)	
٨- الألم عند الوقوف مرتديًا جبيرة تقويم العظام؟ (مثل الجبيرة والأربطة الضاغطة وغيرها)	
٩- ألم القدم عند نهاية اليوم؟	

لا يوجد صعوبة	٠	١	٢	٣	٤	٥	٦	٧	٨	٩	١٠	صعب جدًا وغير قادر

**مقياس الإعاقة او الصعوبة:<br>مدى الصعوبة التي تجدها عند:**	**الدرجة**
١٠- المشي في المنزل؟	
١١- المشي خارج المنزل؟	
١٢- المشي مسافة ٨٠٠ متر؟	
١٣- صعود الدرج؟	
١٤- النزول من الدرج؟	
١٥- الوقوف على طرف الأصابع؟	
١٦- النهوض من على الكرسي؟	
١٧- طلوع الرصيف؟	
١٨- المشي بسرعة؟	

لا شيء من الوقت	٠	١	٢	٣	٤	٥	٦	٧	٨	٩	١٠	كل الأوقات

**مقياس حدود النشاط:**<br>**كم مدة الوقت الذي:**	**الدرجة**
١٩- تقضيه داخل المنزل خلال اليوم بسبب ألم قدميك؟	
٢٠- تقضيه في الفراش خلال اليوم بسبب ألم قدميك؟	
٢١- تَحُدّ فيه من نشاطك بسبب ألم قدميك؟	
٢٢- تستخدم فيه أجهزة المساعدة على المشي داخل المنزل؟ (مثل العصا والعكاز وغيرها)	
٢٣- تستخدم فيه أجهزة المساعدة على المشي خارج المنزل؟(مثل العصا والعكاز وغيرها)	

النتيجة الكلية (/ ٢٣٠)
